# Revealing caprine schistosomiasis and its One Health importance in Malawi: A molecular epidemiological investigation augmented with a praziquantel treatment and GPS animal tracking pilot sub-study

**DOI:** 10.1016/j.onehlt.2024.100918

**Published:** 2024-10-19

**Authors:** Alexandra Juhász, Peter Makaula, Lucas J. Cunningham, Lewis Field, Sam Jones, John Archer, Bright Mainga, David Lally, Gladys Namacha, Donales Kapira, Priscilla Chammudzi, E. James LaCourse, Clinton Nkolokosa, Edmund Seto, Sekeleghe A. Kayuni, Janelisa Musaya, J. Russell Stothard

**Affiliations:** a Liverpool School of Tropical Medicine, Liverpool, UK; b Semmelweis University, Budapest, Hungary; c Malawi Liverpool Wellcome Research Programme, Blantyre, Malawi; d Kamuzu University of Health Sciences, Blantyre, Malawi; e University of Washington, Seattle, WA, USA

**Keywords:** *Schistosoma mattheei*, *Schistosoma haematobium*, Miracidia hatching test, *Capra hircus*, *Bulinus africanus*, Zoonosis

## Abstract

To shed first light on caprine schistosomiasis and its zoonotic potential in Malawi, we conducted a molecular epidemiological investigation, sampling goats (*n* = 230) across three districts, using faecal miracidia hatching test. Molecular genotyping of miracidia later revealed the prevalence of *Schistosoma mattheei* to be 0.0 % in Nsanje District (*n* = 30), 16.7 % in Chikwawa District (n = 30) and 25.3 % in Mangochi District (*n* = 170). Notably, a miracidium of *Schistosoma haematobium* was observed from a single goat in Chikwawa. Inspection of carcasses (*n* = 51) at two local abattoirs in Mangochi District did not find any evidence of caprine schistosomiasis where only a single herd, at Mangochi 3, was infected. Here, despite sampling several other herds nearby, the prevalence was 87.7 % (*n* = 49), with an animal found excreting 1000 miracidia per 5 g of faeces. At this location, our praziquantel treatment (*n* = 14) and GPS animal tracking (*n* = 2) pilot sub-study compared two local goat herds over a three-month period. The daily foraging ranges across a 10 km^2^ area were recorded, alongside targeted schistosome surveillance within local freshwater intermediate snail hosts. Analysis of GPS data revealed only one herd (infected) to have regular daily water contact with Lake Malawi whereas the other herd (not infected) totally avoided the lake. One week after praziquantel treatment administered at 40 mg/kg, anthelminthic cure rate was 92.3 % while at three months approximately a third of treated animals were shedding schistosome miracidia. Cercariae from several field-caught snails locally were genotyped, inclusive of finding a *Schistosoma haematobium*-*mattheei* hybrid. Our findings reveal the focalized nature of caprine schistosomiasis, signposting a novel alert for *S. haematobium* transmission, and highlight where zoonotic transmission can be intense. To better address zoonotic spill-over from *S. mattheei* (and/or *S. haematobium*), the national control programme for schistosomiasis should formally develop targeted surveillance of caprine schistosomiasis and where appropriate, attempt an integrated One Health intervention in future.

## Highlights

• First molecular epidemiological survey of caprine schistosomiasis in Malawi
• Seminal use of GPS data loggers to record the spatial movements of goats along the shoreline of Lake Malawi
• Demonstration of short- and long-term anthelminthic performance of praziquantel in goats administered at 40 mg/kg
• Generates a solid rationale to formally include caprine surveillance within the national control programme for schistosomiasis

## Graphical abstract

**Figure:**
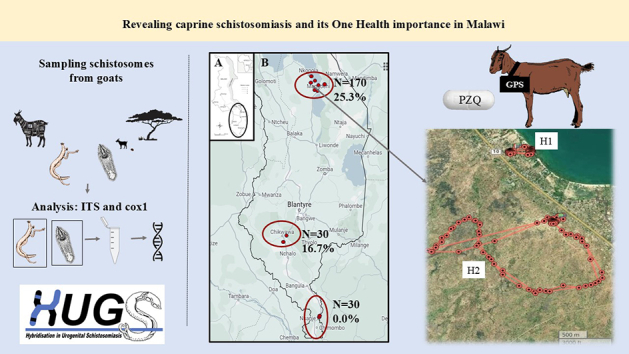

## 1 Introduction

*Schistosoma mattheei* is a multi-host parasitic blood fluke and a confirmed veterinary pathogen for ruminants in Southern Africa, commonly infecting farmed livestock: cattle, sheep and goats [1]. It can also infect humans, inclusive of hybridizing with *Schistosoma haematobium* [[2], [3], [4]]*.* Recently, in West Africa, goats have emerged as significant hosts for zoonotic schistosomiasis, becoming major sources of infection alongside cattle [5]. The increasing role of goats in the spread of schistosomiasis presents challenges for disease prevention and control in endemic regions [6].

Over the last few decades, goat populations have surged in some African regions [7,8], including rural Malawi, where they play crucial roles in food security, supplemental income [9], traditional ceremonies and household financial stability [10,11]. However, such small holdings suffer from animal losses and reduced productivity due to infections with schistosomiasis [12]. Despite the growing recognition of goats as common sources of *S. mattheei* infection, their role in zoonotic transmission has not received adequate attention in Central and Southern Africa [1,6,13]. Given their low maintenance costs and resilience, goats are ideal for small-scale farmers [7,14] although free-range grazing practices likely increase local schistosome infection rates and environmental contamination from infected goat faeces [15].

In light of recent findings of zoonotic schistosomiasis in cattle in southern Malawi [16], it is necessary to clarify if caprine schistosomiasis is equally as important. Here, since contemporary epidemiological information on goats is absent, we sought to estimate the prevalence and transmission dynamics of caprine schistosomiasis across three districts: Nsanje, Chikwawa and Mangochi. In Mangochi District, we later undertook a praziquantel treatment and GPS animal tracking pilot sub-study, inclusive of freshwater snail surveillance, across a three-month study period starting from mid-July 2022.

## 2 Materials and methods

### 2.1 Study area, faecal sample collection and miracidia hatching test

**Fig. 1:**
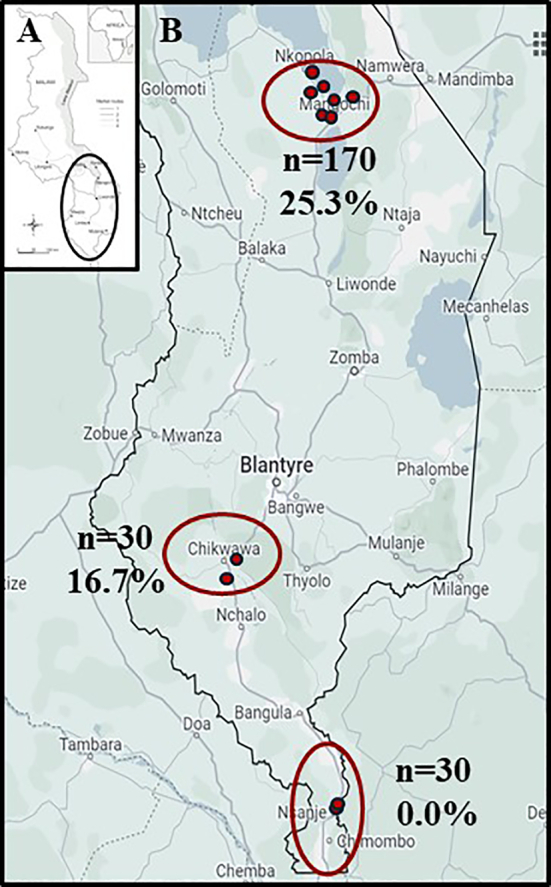
Sampling area for caprine schistosomiasis. A) Map of Malawi; B) Map showing study sites and the prevalence of *Schistosoma* spp. infections in goats sampled from Malawi (Mangochi, Chikwawa and Nsanje Districts), Southeastern Africa from October 2021 to October 2022.

Our study was conducted at eleven inspection sites across Nsanje (*n* = 2), Chikwawa (n = 2) and Mangochi (*n* = 7) districts in southern Malawi (Fig. 1), with sampling undertaken between October 2021 and September 2022. Our study on caprine schistosomiasis forms part of a broader “Hybridisation in UroGenital Schistosomiasis (HUGS)” investigation that is conducting concurrent epidemiological inspections in people and freshwater snails. Initially, our baseline standard sample size was set at 15 animals per location, with duplicate locations sampled per district. In Mangochi District, however, it was later appreciated that certain goat herds entered into Lake Malawi, consequently, we sampled an additional five locations to permit a better local appraisal along this lake's shoreline.

Faecal samples were collected from 230 individual goats from three districts, the majority of faecal samples were obtained per rectum with the remainder picked from the ground as freshly deposited faecal pellets as observed directly upon animal defaecation. The faecal miracidia hatching test (MHT) was used to estimate the prevalence and intensity of infection, as previously described [17], with approximately 5 g of faecal sample examined. Under a dissecting microscope, individual miracidia were harvested onto Whatman FTA® cards (GE Healthcare Life Sciences, Amersham, UK) for later parasite genotyping. If the MHT gave a positive result, the remaining faecal sediment was examined under x100 microscopy for helminth eggs.

### 2.2 Inspection of carcasses at abattoir

To augment faecal analysis in Mangochi District, a total of 51 goat carcasses was examined at two abbatoirs across a working week, Central Market (14.481114°S, 35.261843°E) and ADMARC Market (14.483667°S, 35.249860°E), inspecting mesenteric veins for adult worms alongside conducting MHT on faeces. Attempts were made to ascertain animals' point-of-origin by inspection of slaughter registers, however, no formal written reporting other than the total of animals processed each day were noted.

### 2.3 Molecular identification of schistosomes

Genomic DNA from miracidia stored on Whatman FTA® cards was alkaline eluted for genotyping with a newly described high-resolution melt (HRM) analysis of both nuclear (nDNA) and mitochondrial (mtDNA) marker loci [19]. Adult schistosomes were provided for genetic analysis upon an opportunistic animal autopsy from the praziquantel treatment sub-study, using the HRM assay [19]. Samples of interest (i.e., putative hybrids or human schistosomes) were later confirmed upon Sanger DNA sequencing of sub-regions of the nuclear ribosomal internal transcribed spacer (ITS) and mitochondrial cytochrome oxidase sub-unit 1 (*cox*1), respectively [19,20] and confirmatory re-screen with the mitochondrial ribosomal 16S species specific real-time PCR assay with Taqman® probes [21].

### 2.4 Praziquantel treatment and GPS animal tracking pilot sub-study

**Supplemental Fig. 1:**
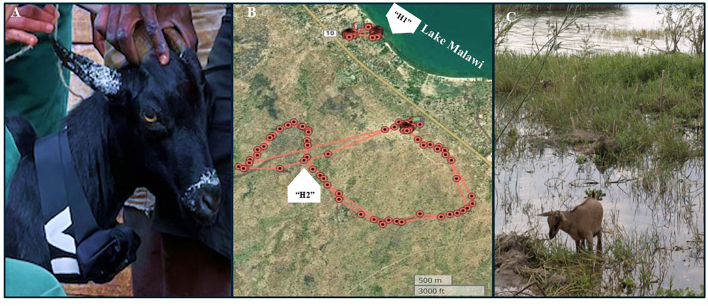
A) Goat fitted with a satellite GPS datalogging unit with nylon collar for animal tracking. B) Satellite image map of the GPS study area showing the initial movement of the two herds (H1,H2) movements (red lines) during a two-day period in early July. This spatial patterning was further repeated during the July to October study period. Goats' movements were very clearly restricted to the areas each day being constantly under guided and under immediate supervision by their local herder. C) Photograph of goat from herd H1, seen browsing on emergent vegetation. Of note infected intermediate snail hosts of schistosomiasis have been found at this site. Moreover, the H1 herd is in regular water contact with this site which is also a local human water contact site for daily washing and swimming.

Upon preliminary findings at Mangochi 3, the movements and relative water contact levels of two targeted goats, as proxy markers of their respective herds, were each recorded over a three-month period (98 days) (20th July 2022 to 16th October 2022) using GPS datalogger technology, see Supplemental Fig. 1A. Two local herds, designated as herd “H1”(*n* = 150) and herd “H2”(*n* = 30) were examined. Both herds were observed to undergo free-roaming during the day although under supervision by different herders. Each night, animals returned to their raised sleeping pens at: H1 (14.36579° S, 35.16994° E) and H2 (14.378329° S, 35.176842° E). Goats were eligible for sub-study inclusion if they were not regularly tied or enclosed, were at least six months old and were not planned for slaughter within the next three months.

One goat from each herd was selected for GPS tracking. In herd H1, 14 further animals were fitted with nylon harness with green plastic medallions (*n* = 1–14), each medallion was labelled on both sides with indelible ink. Both GPS study goats were tracked over the same three-month period, and GPS data were downloaded following methods described in [19]. The analysis of GPS data was conducted using R software (version 4.3.1) and QGIS 3.22.1, to estimate the locations and water contact times of the two animals, as proxies of their respective herds. After first perusal of GPS track logs, see Supplemental Fig. 1B, a selection of GPS points were verified on-the-ground to corroborate confirmed water contacts by visual inspection, see Supplemental Fig. 1C.

Fresh faecal samples were collected from the rectum of 15 adult H1 goats and the GPS fitted goat from Herd H2, at three inspection points (baseline, one week and 12 weeks). Bodyweight estimates were obtained using chest/heart girth measurements [22] to calculate the required praziquantel (600 mg IDA Pharmacy) dosage (40 mg/kg) for oral administration on 23rd July 2022 by the lead veterinarian (AJ), after baseline collection of faeces. Although this dosage is recommended by World Health Organization (WHO) guidelines [23], we were aware other dosages, ranging from 20 to 60 mg/kg, are used on goats elsewhere [[24], [25], [26]].

### 2.5 Surveillance of freshwater intermediate snail hosts

In July and October 2022, two freshwater snail surveys were conducted to assess the abundance of *Schistosoma* spp. within intermediate snail hosts in the local vicinity of the GPS studied area. Snails were subjected to cercarial shedding analysis, with schistosome cercariae collected onto FTA® cards for later HRM analysis, with snail identity confirmed by sequence analysis of the *cox*1 gene [27]. Prepatent infections were assessed upon extracting snail genomic DNA then screened with a real-time PCR assay with genus-specific schistosome TaqMan® probe [29].

### 2.6 Research ethics approvals

Research was approved by College of Medicine Research Ethics Committee (COMREC) of Kamuzu University of Health Sciences (KUHeS) in Malawi (approval no. P.08/21/3381) and Liverpool School of Tropical Medicine Research Ethics Committee (LSTM-REC; approval no. 22–028). Praziquantel treatment was judged standard of care for animal welfare reasons.

## 3 Results

### 3.1 Analysis of goat faeces and carcasses

**Fig. 2:**
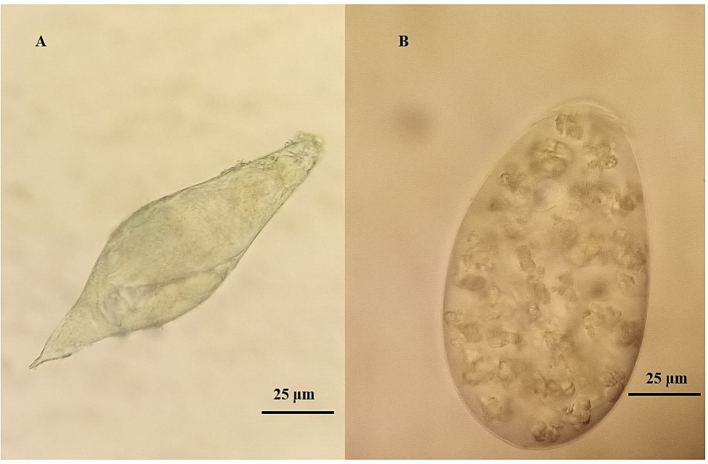
Parasite eggs recovered in faecal samples from goats by sedimentation. A) *Schistosoma mattheei* egg and B) rumen fluke egg, each photographed at 400× by light microscopy, no stain used.

The MHT confirmed caprine schistosomiasis in two of three districts, with an overall prevalence of 20.9 % and highest infection prevalence at Mangochi 3 (Table 1). Upon faecal sedimentation analysis, typical eggs of *S. mattheei* were observed often together with rumen fluke eggs (Fig. 2A & B).

**Table 1:** Prevalence (%) of *Schistosoma mattheei* in faecal samples of goats across 11 study sites.

	Coordinates		Goat		Miracidia no. (in 3 mins)		*Schistosoma* sp.
Study site	South	East	N _MHT_⁎	No. positive	Max.	Mean	Prevalence (%) 95 % CI
Mangochi 1	14.48086°	35.26242 °	40	0			0
Mangochi 2	14.48309°	35.25023°	11	0			0
Mangochi 3^	14.36579°	35.16994°	49	43	1000	26.72	87.7 (75.2–95.4)
Mangochi 4	14.37833°	35.17684°	20	0			0
Mangochi 5	14.44942°	35.23875°	20	0			0
Mangochi 6	14.37117°	35.14174°	15	0			0
Mangochi 7	14.47082°	35.27946°	15	0			0
Total from Mangochi			170	43			25.3 (18.9–32.5)
Nsanje 1	16.85401°	35.29956°	15	0			0
Nsanje 2	16.92376°	35.25773°	15	0			0
Total from Nsanje			30	0			0
Chikwawa 1	16.10088°	34.42346°	15	5	3	7	33.3 (11.8–61.6)
Chikwawa 2	16.09739°	34.83376°	15	0			0
Total from Chikwawa			30	5			16.7 (5.6–34.7)
Total examined			230	48		16.86	20.9 (15.8–26.7)

⁎ Number of goat sampled by miracidia hatching technique.

^ Mangochi 3 also included later sampling of Herd 1.

Sampling 51 goat carcasses at the abattoirs in the Mangochi District did not detect any evidence of schistosome infection. The autopsy examination of goat 2 from herd H1, however, yielded approximately 50 unpaired adult worms within the hepatic portal vein, which were later genotyped by HRM and DNA sequencing.

### 3.2 Genotyping miracidia and adult worms by HRM analysis

**Fig. 3:**
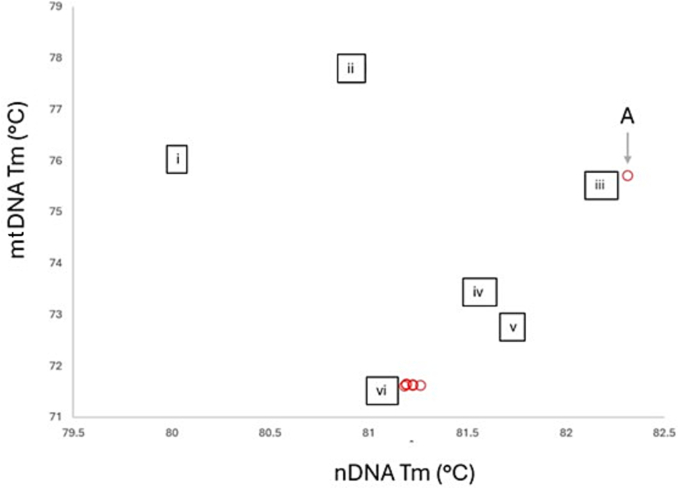
Genotyping miracidia and adult worms. Bivariate scatter plot denoting the mtDNA and nDNA melt-peaks (Tm) results for the two tube HRM assay. Square markers denote positive controls for i) *S. mansoni*, ii) *S.margrebowiei*, iii) *S. haematobium*, iv) *S. bovis*, v) *S. curassoni* and vi) *S. mattheei*. The position of one pure *S. haematobium* found within a goat from Chikwawa is study site 1 shown with labels “A”.

Two hundred miracidia on Whatmann FTA® cards, originating from 46 goats, were processed from which 106 yielded nDNA amplicons and 101 yielded mtDNA amplicons, generating a combined data set for mtDNA and nDNA markers for 88 miracidia. Of these, 87 (98.9 %) clustered with the expected melt temperatures (Tm) of *S. mattheei* but one sample (1.1 %), from Chikwawa, clustered with the expected Tm of *S. haematobium* (Fig. 3). This *S. haematobium* positive sample was further confirmed using the mitochondrial 16S species-specific TaqMan® probe assay.

In total 23 males and 23 females obtained from goat 2 within herd H1 were screened using the HRM assay. All worms were genotyped as *S. mattheei*. As further confirmation a ribosomal ITS and *cox*1 sequence were each obtained and upon BLAST search matched *S. mattheei*.

### 3.3 Snail surveillance and analysis of cercariae

In July 2022, along the shoreline of Mangochi 3, no collected *Bulinus* spp. snail was observed to shed schistosome cercariae, however, molecular xenomonitoring revealed seven out of 90 snails with pre-patent infections, which upon HRM analysis evidenced a snail with *S. mattheei* and another with *S. haematobium* infections. Snail identification upon inspection of the *cox*1 sequence and BLAST search of GenBank matched *Bulinus africanus* accession number AM286296.2. In October 2022, after a repeat survey, three *B. africanus* were found shedding schistosomes. Of the 16 cercariae screened by HRM, seven were characterised at both nDNA and mtDNA loci: five were *S. mattheei*, while two were introgressed hybrids being typed as *S. haematobium* for mtDNA and *S. mattheei* nDNA.

### 3.4 Praziquantel treatment and GPS animal tracking pilot sub-study

The infection status of two herds was previously confirmed (see Table 1), 13 out of 15 selected animals (86.7 %) in herd H1 tested positive for *Schistosoma* spp. (Table 2). In contrast, herd H2 was negative for *Schistosoma* spp. at all inspection timepoints by MHT. One week post-treatment, praziquantel demonstrated satisfactory efficacy with overall prevalence reducing from 86.7 % to 14.3 %, an anthelminthic cure rate of 92.3 % with substantial reductions in numbers of miracidia observed (Table 2). At 12 weeks inspection, 33.3 % of the examined animals tested positive for MHT with miracidia observed in low numbers. During this time both goat herders continued with their daily routine and herding duties as before.

**Table 2:** Infection status of goats in herd H1, before- and after treatment (1 week) with miracidia hatching results.

	Pre-treatment infectious status (miracidia count/3 mins)	Post-treatment infection status (miracidia count/3 mins)
Goat ID	Baseline	1-week
GPS I	positive (10)	positive (1)
1	positive (23)	negative
2	positive (1)	n.a. ⁎
3	positive (7)	negative
4	positive (23)	negative
5	positive (1)	negative
6	positive (1000)	negative
7	positive (2)	negative
8	positive (2)	negative
9	positive (16)	negative
10	positive (7)	negative
11	negative	negative
12	positive (180)	positive (6)
13	positive (10)	negative
14 (untreated control)	negative	positive (2)

⁎ goat 2 was autopsied after death and yielded several adult schistosome worms.

**Fig. 4:**
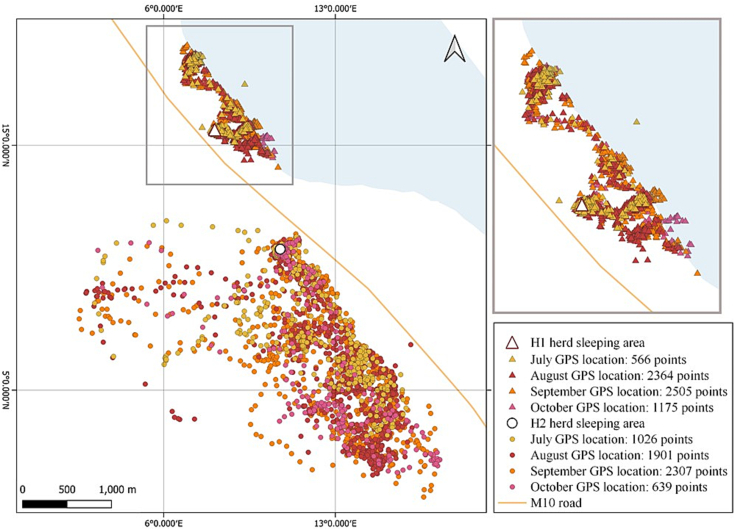
Plot of goat movements across the GPS tracking pilot sub-study area. Monthly GPS traces from the herd H1 and H2 from the July 2022 to October 2022 period. The snail collection site of Mangochi 3 is a ∼200 m section of the shoreline, along which H1 herd roamed, and is described in greater detail in [28].

Over the 3-month period a total of 6610 and 5873 GPS cordinates recording the goats position were collected for H1 and H2 animals, respectively. These individual co-ordinates are plotted for each animal by month (Fig. 4). From analysis of these data, goat H1 had a cumulative water contact exposure period of 13.75 h whereas goat H2 had no water contact exposure during this period (Fig. 4). Although there was some day-to-day variation in water contacts, the average period of exposure across the study was approximately 15 min per day for goat H1.

## 4 Discussion

Our study is, to our knowledge, the first formal survey of caprine schistosomiasis in southern Malawi, implementing faecal examination with MHT, and carcass inspections, to detect and to characterise schistosome infections. We have provided clear evidence of *S. mattheei*, and uniquely the seminal presence of *S. haematobium* within our sampled animals. Our findings add to wider concerns about *S. mattheei*'s potential for increased transmission and hybridization with *S. haematobium* which could influence both animal and human health, and impact upon livestock economics [16,18].

From our associated freshwater snail surveillance, the detection of *S. mattheei*, as well as *S. haematobium*, shedding from *B. africanus* in Lake Malawi has not been reported before. It was previously assumed that only *Bulinus globosus* was present in this area [[28], [29]], however, application of molecular methods of identification and xenomonitoring has been insightful. As an additional note on intermediate host snails, in the inland area visited by goat herd H2 we later identified populations of *B. forskalii* in ephemeral pools away from the lake. Though this snail species can act as a host of various schistosomes [[30], [31], [32]] and more recently been implicated in transmission of *S. haematobium* (and or its associated hybrids) in Niger and Senegal [33,34], we found no evidence of transmission here and do not consider this species of local importance.

### 4.1 Schistosome environmental contamination and infection pathways

**Fig. 5:**
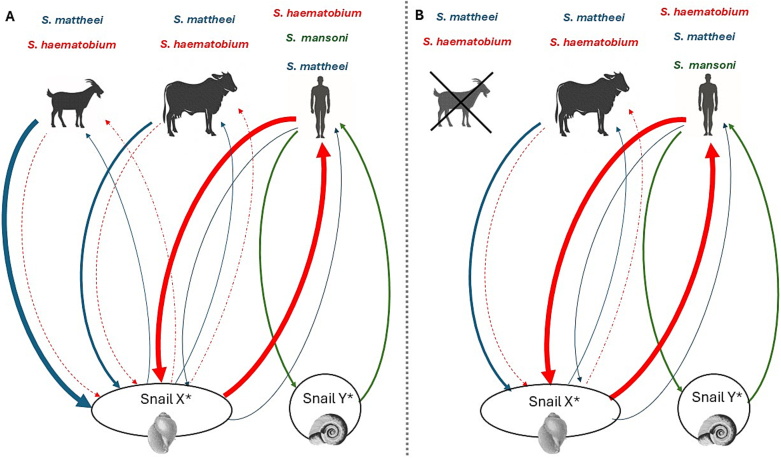
A visual hypothesis of how (overlapping) transmission pathways that might connect caprine schistosomiasis to human and bovine schistosomiasis and highlight the need to carefully identify which livestock should be considered within future targeted control. A) Where infected goats are present these will contaminate local species of *Bulinus* (species A), however, such infected snails may incubate *S. mattheei* and then release cercariae that infect both bovines and people. Other schistosomes that originate from bovines and cattle might lead to infections in goats. B) Where infected goats are not present, schistosome transmission pathways are simplified and would imply that control is not needed.

Our better appreciation of zoonotic schistosomiasis, and its pathways in local freshwater snails, is attempted in schematic in Fig. 5. Goats in southern Malawi, particularly in herd H1, that graze daily within Lake Malawi's shoreline or in Chikwawa 1 that graze along the Mwanza River's margins, can have very high (∼85 %) to moderate (∼25 %) schistosome infection levels. However, many other nearby herds remain free from exposure and infection. Although such infected herds remain to be precisely identified and more fully quantified, it is appropriate to assume that they presently contribute towards tangible levels of environmental contamination with schistosome miracidia of *S. mattheei* (and potentially *S. haematobium*). While cattle and goats usually graze together in *Schistosoma*-infected areas, compared to cattle, some infected goats appear to release much more miracidia per gram of faecal, ∼ 3.37 for goats and ∼ 0.26 for cattle, even though their total amount of faecal production per animal is substantially less, as is their water contact exposure times about 15 min per day versus about 35 min for cattle [16]. Future experimentation comparing cattle and goats in laboratory-controlled infection and treatment studies could be particularly informative in clarification of the relative importance of each livestock's potential role in zoonotic schistosomiasis.

The higher number of goats compared to cattle in certain regions can be attributed to their smaller body size and lower daily water intake [7]. Due to their reduced body mass, goats require less food and water than cattle, making them more economical to raise in environments where resources are scarce [10]. Additionally, the smaller size of goats makes them less expensive to treat and manage, further reducing the overall cost of care compared to larger livestock like cattle [8,9]. Therefore, to reduce the risk of human and bovine schistosomiasis in endemic areas, it is worthwhile to focus on the treatment of caprine schistosomiasis when needed (Fig. 5A). Targeting goats in treatment programmes could therefore be essential in disrupting the transmission cycle and reducing the prevalence of zoonotic schistosomiasis and bovine schistosomiasis in key foci but might not be needed in others where goats are not common or are considered absent (Fig. 5B).To detect zoonotic schistosomiasis effectively, it is crucial to identify areas where such infected animals regularly contact water and at these sites engage in dialogue with animal herders, encouraging the exploration of alternative watering sites, particularly those away from water contact sites used by people, alongside provision of appropriate communcal watering troughs for livestock.

### 4.2 Impact of grazing behavior and water access: GPS tracking insights

The use of GPS animal tracking devices adds a novel dimension to the epidemiology and future control of schistosomiasis in livestock, enabling a direct comparison between different environments and their associated risks of schistosomiasis exposure, alongside risk of reinfection after treatment, through water contact. Analysis of our GPS data indicated daily water contact for one herd with Lake Malawi, resulting in infection, while the other herd had no observable contact with the lake water, despite its proximity, and remained not infected (Fig. 5). This underscores the importance of micro-environmental factors and human herding behaviours in caprine schistosomiasis transmission. The goat movement analysis indicates that the herd H1 had substantial lake water contact, particularly in the morning hours (8–10 am). The increase in lake water contact points and an associated cumulative exposure time increase during September suggests a possible seasonal or environmental factor influencing behavior (Fig. 5). On the contrary, the absence of lake water contact in the H2 herd could be due to movement patterns influenced by human herding practices (i.e., avoiding crossing the M10 highway were speeding traffic is common place) and greater availability of vegetation for animals to browse upon. Goats are able to access water by browsing on trees and shrubs, unlike cattle, which primarily graze on grasses and require direct water sources. This ability allows goats to survive in arid environments and affects vegetation dynamics by influencing plant community composition [35].

### 4.3 Praziquantel treatment efficacy and control challenges

Our praziquantel treatment sub-study sheds first light on the performance of this anthelminthic within a Malawi caprine setting. The dosage was selected based on the WHO guidelines for our animals at 40 mg/kg [23], this reduced prevalence of active schistosomiasis but did not achieve a 100 % intial parasitological cure, likely due to a combination of hyper-infection status alongside daily water contact that continues the flow of cercariae and later maturing worms into these animals. Praziquantel is ineffective against juvenile stages of this parasite, meaning that very early infections present at the time of treatment would not be eradicated. As shown in Table 2, the efficacy of this treatment is demonstrated by the reduction in miracidia counts, with at least a 95 % reduction observed in the goats treated. The total number of miracidia counts decreased from a baseline of 1281 to 9, one week after treatment indicating a significant knockdown effect on infections.

Perusal of these results might suggest that while the treatment was effective for the majority of the herd, a small subset of goats did not respond completely, indicating potential variability in drug response or infection severity. The pharmacokinetic-pharmacodynamic profile of praziquantel for caprine schistosomiasis, particularly for *S. mattheei* infections, is not extensively documented in the literature, highlighting a need for further research to optimize raised dosing regimens [[24], [25], [26]]. In goats, the required dosage can range from 20 to 60 mg/kg, depending on the severity of the infection and the specific pharmacokinetic characteristics of the drug. For an average-sized goat weighing 25 kg, the required total dose of praziquantel is 500 mg, 1000 mg or 1500 mg for dosages of 20 mg/kg, 40 mg/kg, or 60 mg/kg, respectively, necessitating the administration each of approximately 1, 2 or 2.5 tablets (i.e. 600 mg with a half score division) crushed and suspended into ∼200 ml water with bottle-fed delivery. Once the goat has been restrained this treatment procedure takes no longer than 5 min per animal. Here in Malawi, whilst this demand for praziquantel for each animal in veterinary medicine might appear modest, as are the appropriate animal handling skills to do so, there is no commercial veterinary supply nor are livestock veterinarians/owners specifically trained in such bottle-based deworming.

### 4.4 Integrating caprine schistosomiasis management into the national programme

For interruption and elimination of environmental transmission of schistosomiasis, it will become evermore important to integrate caprine schistosomiasis management into Malawi's national control programme. To do so, we encourage dialogue with the Animal Health Directorate and establish an effective One health approach [16,36]. Nonetheless, effective prevention and control strategies face significant challenges, including inadequate diagnostic/detection methods, bottlenecks in praziquantel supplies and the lack of effective animal vaccines. Spatially targetted prevention and treatment plans as based on accurate local epidemiological knowledge, are necessary, especially directed investigations and routine surveillance of free-ranging goats in infected snail-infested areas. Sustainable development paths combining goat farming with schistosomiasis control, intensive breeding, and the reduction of *Bulinus* spp. in susceptible areas are beneficial [37]. To better address zoonotic spill-overs from *S. mattheei* (and/or *S. haematobium*), for example, the Malawi national control programme for schistosomiasis should work closely with the veterinary sector to develop then include targeted surveillance with later attempts in integrated control within such infected livestock herds.

## 5 Conclusions

Our study has documented the presence of caprine schistosomiasis caused by *S. mattheei* in goats, focusing future attention within Mangochi and Chikwawa Districts, alongside signposting a public health concern of *S. haematobium* transmission potential. We have demonstrated that praziquantel treatment at a dosage of 40 mg/kg, whilst generally effective, does not achieve an absolute short-term cure and, with reinfection, some third of treated animals are infected after 12 weeks. Caprine schistosomiasis in goats is therefore an important but overlooked veterinary and public health problem in Malawi, we strongly encourage collaborative efforts among key stakeholders such as veterinarians, livestock owners, public health officials and researchers to combat this parasitic disease and improve both animal welfare and human health.

The following is the supplementary data related to this article.

## Funding

The Hybridisations in UroGenital Schistosomiasis (HUGS) project receives funding from the 10.13039/100010269Wellcome Trust (220818/Z/20/Z).

## CRediT authorship contribution statement

**Alexandra Juhász:** Writing – review & editing, Writing – original draft, Methodology, Investigation, Formal analysis, Data curation. **Peter Makaula:** Writing – review & editing, Writing – original draft, Methodology, Investigation, Formal analysis. **Lucas J. Cunningham:** Writing – review & editing, Writing – original draft, Methodology, Investigation, Formal analysis. **Lewis Field:** Writing – review & editing, Writing – original draft, Methodology, Investigation. **Sam Jones:** Writing – review & editing, Writing – original draft, Methodology, Investigation, Formal analysis, Data curation. **John Archer:** Writing – review & editing, Writing – original draft, Methodology, Investigation. **Bright Mainga:** Writing – review & editing, Writing – original draft, Methodology, Investigation. **David Lally:** Writing – review & editing, Writing – original draft, Methodology, Investigation, Data curation. **Gladys Namacha:** Writing – review & editing, Writing – original draft, Methodology, Investigation. **Donales Kapira:** Writing – review & editing, Writing – original draft, Methodology, Investigation. **Priscilla Chammudzi:** Writing – review & editing, Writing – original draft, Methodology, Investigation. **E. James LaCourse:** Writing – review & editing, Writing – original draft, Supervision, Methodology, Investigation. **Clinton Nkolokosa:** Writing – review & editing, Writing – original draft, Visualization, Software, Methodology, Formal analysis. **Edmund Seto:** Writing – review & editing, Writing – original draft, Visualization, Software, Methodology, Formal analysis. **Sekeleghe A. Kayuni:** Writing – review & editing, Writing – original draft, Supervision, Project administration, Methodology, Investigation. **Janelisa Musaya:** Writing – review & editing, Writing – original draft, Validation, Supervision, Project administration, Methodology, Investigation, Funding acquisition, Conceptualization. **Russell Stothard:** Writing – review & editing, Writing – original draft, Validation, Supervision, Project administration, Methodology, Investigation, Funding acquisition, Conceptualization.

## Declaration of competing interest

The authors declare that they have no known competing financial interests or personal relationships that could have appeared to influence the work reported in this paper.

## Data Availability

Data will be made available on request.
